# Cost-effectiveness of robotic-assisted versus conventional total knee arthroplasty: an analysis from a middle income country

**DOI:** 10.2340/17453674.2025.44753

**Published:** 2025-09-30

**Authors:** Pakpoom Ruangsomboon, Onlak Ruangsomboon, Wanrudee Isaranuwatchai, Michael G Zywiel, David MJ Naimark

**Affiliations:** 1 Sunnybrook Health Sciences Centre, Division of Orthopaedic Surgery, Toronto, ON, Canada; 2 Faculty of Medicine, Siriraj Hospital, Mahidol University, Thailand; 3 Institute for Health Policy Management and Evaluation, Dalla Lana School of Public Health, University of Toronto, Toronto, ON, Canada; 4 Health Intervention and Technology Assessment Program (HITAP), Department of Health, Ministry of Public Health Tiwanon Road, Muang District, Nonthaburi, Thailand; 5 Division of Orthopaedic Surgery, University Health Network, Toronto Western Hospital, Toronto, ON, Canada; 6 Upstream Lab, MAP Centre for Urban Health Solutions, Li Ka Shing Knowledge Institute, Unity Health Toronto, Toronto, ON, Canada; 7 Division of Nephrology, Department of Medicine, University of Toronto, Toronto, ON, Canada; Statement of location: Our work was performed in all 7 institutions.

## Abstract

**Background and purpose:**

Robotic-assisted total knee arthroplasty (RATKA) can enhance surgical precision. In middle-income countries (MICs), constrained fiscal space and the double burden of rising demand for high-cost technologies and competing public-health priorities—unlike high-income countries with broader fiscal headroom and low-income countries with limited adoption of expensive innovations—make adoption decisions for RATKA particularly challenging. We aimed to evaluate the cost-effectiveness analysis (using a cost-utility framework) of RATKA vs conventional TKA (COTKA) from a societal perspective in Thailand as a MIC.

**Methods:**

A discrete event simulation model was employed to compare the cost-effectiveness of unilateral RATKA with COTKA over 4.5 years from a societal perspective, using patient-level data from January 2018 to June 2022 from an arthroplasty center in Thailand. Patients were propensity matched to balance comorbidities. Base case analysis assumed 1 robot performs 434 TKA cases per year with an anticipated lifespan of 12.5 years. We considered direct medical, non-medical, and indirect costs, alongside quality-adjusted life years (QALYs) gained from a societal perspective. We calculated incremental net monetary benefits (INMB) and cost-effectiveness ratios (ICERs) as the main outcome measures. Sensitivity analyses and 10 scenario analyses were performed exploring various possible settings. Threshold analyses determined combinations where RATKA could be cost-effective with positive INMB under the Thai cost-effectiveness threshold of US$4,888 per QALY gained.

**Results:**

The base case analysis involved 157 COTKA and 1570 RATKA matched cases with a mean age of 69 (standard deviation 8 years). The lifetime average outcomes per patient were: COTKA—US$5,031.9 and 9.07 QALYs; RATKA—US$5,666.9 and 9.16 QALYs. The incremental (RATKA–COTKA) differences were +US$633.6 (95% credible intervals [CrI] ~592–675) and +0.085 QALYs (CrI ~0.04–0.13), yielding an ICER of US$7,436.6/QALY. RATKA was not cost-effective compared with COTKA, with an INMB of –216.9 US$/patient. The probability of RATKA being cost-effective at the Thai cost-effectiveness threshold was 44.3%. For RATKA to be economically attractive, 1 robot must operate on at least 640 TKA cases/year over 12.5 years. 3 scenarios found RATKA to be cost-effective: (i) maximal robot utilization (850 cases/year); (ii) lowest capital costs (611,060 US$/robot) with high efficacy for RATKA (hazard ratio [HR] 0.6); and (iii) extreme efficacy of RATKA in reducing complications (HR 0.024).

**Conclusion:**

In the context of MIC, a broad adoption of RATKA is not economically attractive as treatment of end-stage knee osteoarthritis patients compared with COTKA.

Conventional total knee arthroplasty (COTKA) is the treatment of choice for patients with knee osteoarthritis who have failed nonoperative treatment. Although that survivorship is high [1,2], dissatisfaction rates at 10–15% highlight the potential for further improvements in TKA [3,4].

Advanced technology, such as robotics, has become widely integrated into surgical practices in many fields [5-7]. Robotic-assisted total knee arthroplasty (RATKA) is noted for its precision, as evidenced by its superior radiographic outcomes [6]. Nevertheless, clinical and functional outcomes have not demonstrated superiority over those of COTKA, with inadequate evidence on complication rates or ability to decrease reoperation rate [6,8,9].

From an economic perspective, RATKA introduces significant financial burdens related to robotic equipment, software licensing, maintenance, and training. Previous cost-utility analyses have found that RATKA may be cost-effective in high-volume centers based on a cost-effectiveness threshold of US$50,000/quality-adjusted life years (QALYs) [10,11]. Economic evaluations of RATKA vs COTKA are still scarce and have predominantly been conducted in high-income countries (HICs) [10,11], leaving a gap of evidence from middle-income countries (MICs) defined by the World Bank as those with a gross national income per capita between US$4,096 and US$12,695 [12]. MICs often struggle with challenges such as inequitable access to health insurance and varying levels of health service quality compared with HICs [13,14]. Unlike HICs with broader health coverage or low-income countries with minimal adoption of high-cost technologies, MICs face the dual challenge of rising expectations for advanced care and fragmented health financing systems. The economic implications to implement this high-cost technology within limited budgets in MICs necessitate an economic evaluation to ensure that health resources are distributed effectively [15]. Therefore, we conducted a simulation-model-based economic evaluation to assess the cost-effectiveness (using a cost-utility framework) of RATKA vs COTKA from a societal perspective in Thailand, which is a MIC.

## Methods

We performed a simulation-model-based economic evaluation to assess the cost-effectiveness of a strategy to provide RATKA to all patients with end-stage knee osteoarthritis who have failed nonoperative therapy relative to a strategy of providing COTKA to all such patients. Our economic evaluation adhered to the CHEERS statement [16] (Supplementary Table 1).

### Simulation model structure

We constructed an individual-level discrete event simulation (DES) model using Treeage Pro 2023 (Healthcare Version R1.2, Williamstown, MA, USA) to trace the clinical course of individuals from the initial knee surgery until either death or attaining 110 years of age. We sampled characteristics for each simulated individual as described below. For each individual, we simulated their clinical course subsequent to RATKA and then repeated their simulation after COTKA. The model structure is provided in Supplementary Figures 1.1 to 1.11. In a DES model, individuals move from one discrete event to another in time steps of variable duration. Time step lengths were sampled from parametric time-to-event distributions and measured in continuous time [17]. Health outcomes of orthopedic events modeled to occur after the index surgery included aseptic loosening (including associated periprosthetic fractures), acute periprosthetic joint infection (aPJI), chronic periprosthetic joint infection (cPJI), and revision surgeries. These events were identified using diagnostic and procedural codes from the SiData+ database, following Thai clinical coding standards. Specifically, aseptic loosening was defined using ICD-10-TM code T84.03, and included mechanical failure or periprosthetic fracture not attributed to infection. PJI was identified by ICD-10-TM code T84.5, and further classified as acute if it occurred within 90 days postoperatively, and chronic if beyond 90 days. Revisions were identified by ICD-9-CM code 81.53 (revision of knee replacement) and encompassed all causes excluding periprosthetic fractures, which were analyzed under aseptic loosening. Secondary complications included any further surgeries or reoperations resulting from failed treatment of aseptic loosening, aPJI, or cPJI. During an individual’s simulation in each strategy, accrued costs and QALYs were discounted at an annual rate of 3.0% as per Thai Health Intervention and Technology Assessment Program (HITAP)’s guideline [18].

### Model inputs

**Figure 1:**
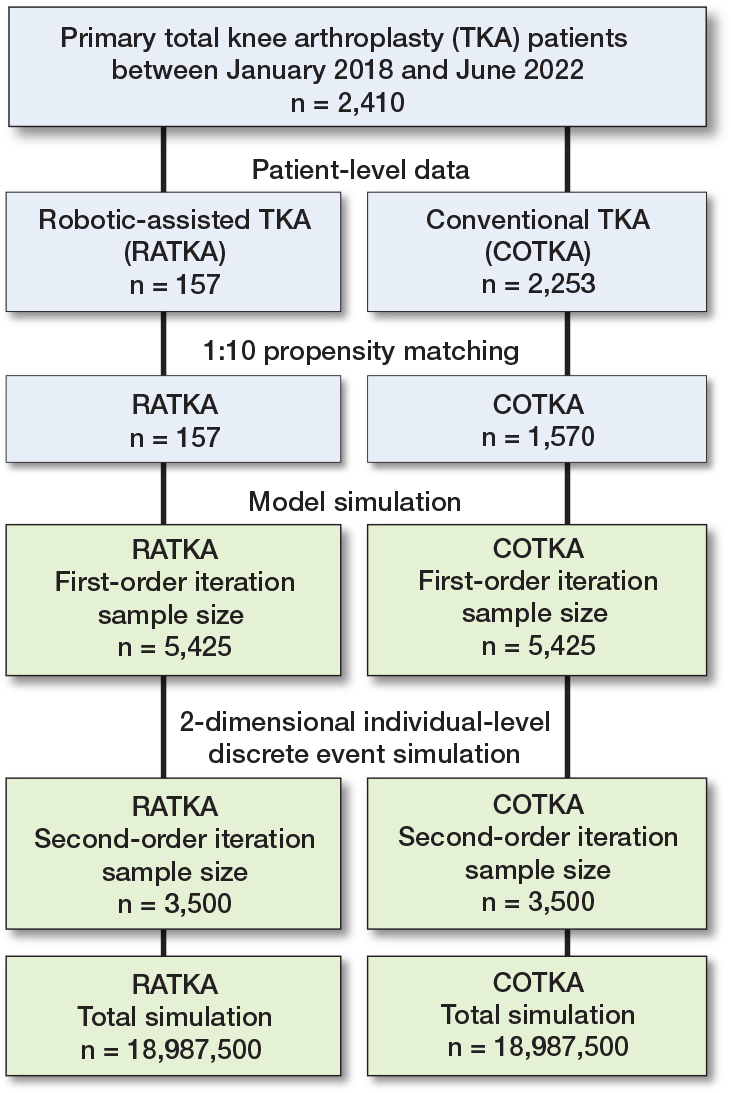
Flow diagram of the study.

Model input variables included age, sex, comorbidities that affect complications after TKA, and type of complications. Time to complication after index surgery [19] was derived from an orthopedic database maintained by Siriraj Informatics and Data Innovation center (SiData+) at Siriraj Hospital, Faculty of Medicine, Mahidol University. Prior to estimating model inputs from the SiData+ registry, we employed propensity score matching (PM) to minimize selection bias between patients undergoing RATKA and COTKA. A logistic regression model was constructed to estimate the probability of receiving RATKA as a function of baseline covariates: age, sex, and comorbidities associated with post-TKA complications (including diabetes mellitus, metastatic cancer, depression, Alzheimer’s disease, Parkinson’s disease, chronic kidney disease, obesity, pulmonary disease, and cardiovascular disease). Matching was conducted using a nearest neighbor algorithm with a caliper of 0.2 standard deviations of the logit of the propensity score. A 1:10 matching ratio (RATKA:COTKA) was chosen to optimize sample size while maintaining acceptable covariate balance. Standardized differences and hypothesis testing confirmed balance post-matching (Supplementary Table 6). The matched cohort was used for subsequent model input derivation, including rates of complications after surgery (Supplementary Table 7). Input data for the simulation model was drawn from the propensity-matched cohort (Figure 1). Lists of model input variables are provided in Supplementary Tables 2–4. Missing data were minimal in the SiData+ registry, with fewer than 5 cases missing for any given variable across the study period (January 2018 to June 2022). In compliance with hospital privacy protocols, if any categorical variable had fewer than 5 members, the value was suppressed and reported as “< 5.” No imputation was performed, and the complete case analysis assumption was deemed reasonable under these conditions. For costs in the base-case analysis, we adopted a societal perspective. Direct medical costs were derived from the hospital’s case-costing database maintained by SiData+, which includes itemized patient-level costs such as implants, surgical instruments, medications, anesthesia, inpatient nursing care, radiology, laboratory investigations, and rehabilitation services. Direct non-medical costs, such as patient travel, food, and informal caregiver time, were estimated using the Thai standard cost list [20]. The diminished or lost capacity to work or participate in leisure activities was reflected as a disutility penalty for simulated individuals [21]. Cost data are reported in US dollars converted from Thai Baht (THB) from the price year 2020 (32.730 THB to 1 US$ as of March 31, 2020, https://www.exchangerates.org/exchange-rate-history/usd-thb-2020-03-31). A list of the cost inputs to the model is provided in Supplementary Table 2. Preference weights (utilities) for health states were derived in this study by mapping EQ-5D-5L responses of patients in the PM cohort to utility values [22] (Supplementary Table 3). Other utility values were obtained from the literature (Supplementary Table 4). To account for parameter uncertainty, model inputs were assigned appropriate probability distributions (e.g., Gamma, Beta, Bernoulli, Normal, Truncated Poisson, Weibull) as detailed in Supplementary Tables 2–4.

### Study population, real-world patient characteristics, and propensity matching for model parameter estimation

The orthopedic database housed at Siriraj Hospital comprised 2,410 unilateral primary TKA cases from January 1, 2018, to June 1, 2022; 157 underwent RATKA and 2,253 underwent COTKA. The mean age was comparable in the RATKA and COTKA groups (69.7 vs 69.1 years, respectively; P = 0.3). After PM at a 1:10 ratio (157 RATKA and 1,570 COTKA cases) baseline differences in characteristics between the 2 groups were resolved (Supplementary Table 6). In the PM cohort, the median follow-up duration after the initial surgery was 1,222 days (interquartile range 1,022 days).

### Complications and mortality after surgery

In the pre-PM dataset, there were 17 aseptic loosening, 34 aPJI, and 8 cPJI, all occurring within the COTKA cohort except for 1 aPJI in the RATKA cohort. After PM, there were 7 aseptic loosening, 15 aPJI, and 4 cPJI remaining within the COTKA group, and 1 aPJI in the RATKA group (Supplementary Table 7). There was no mortality event among the patient cohort during the follow-up period. This PM cohort was employed to estimate the patient-level model inputs (Table 1).

**Table 1:** Summary characteristics and incremental results of the base case and scenario analyses

Scenario type, RATKA annual cases	Key model input parameters			Model results					
	Start age, years (SD)	CC ^a^ (M US$)	HR	Occurrence of AL, aPJI, cPJI (times)	Incremental costs (US$, mean [SE])	Incremental Ieff (QALYs, mean [SE])	ICER (US$/QALY)	INMB (US$)	Proposed summary
Base case analysis									
Societal perspective, 434 cases/year	69 (8)	2.19	0.963	27, 43, 16	633.6 (21.0)	0.085(0.023)	+7,436.5	–217.1	Not cost-effective
Scenario analyses									
(1) No adjustment for public/private usage, 434 cases/year	69 (8)	2.80	0.963	27, 43, 16	701.4 (20.5)	0.111 (0.024)	+6,320.0	–158.9	Not cost-effective
(2) Maximum robot usage, 850 cases/year	69 (8)	2.19	0.963	27, 43, 16	400.3 (20.4)	0.103 (0.023)	+3,876.0	+104.6	Cost-effective
(3) Younger patients, 434 cases/year	55 to 65	2.19	0.963	27, 43, 16	577.0 (20.5)	0.106 (0.029)	+5,436.0	–58.1	Not cost-effective
(4) Older patients, 434 cases/year	65 to 75	2.19	0.963	27, 43, 16	620.3 (20.5)	0.123 (0.023)	+5,040.7	–18.7	Not cost-effective **^b^**
(5) Best plausible for RATKA, 434 cases/year	69 (8)	0.61	0.600	17, 27, 10	159.6 (20.4)	0.165 (0.024)	+965.0	+649.2	Cost-effective
(6) Worst plausible for RATKA, 434 cases/year	69 (8)	6.11	0.975	27, 44, 17	1,366.4 (20.7)	0.119 (0.024)	+11,436.6	–782.3	Not cost-effective
(7) Extreme benefit for RATKA, 434 cases/year	69 (8)	2.19	0.024	1, 1, 0	124.0 (21.0)	0.268 (0.024)	+461.7	+1,188.4	Cost-effective
(8) Extreme harm for RATKA, 434 cases/year	69 (8)	2.19	41.7	1,175, 1,879, 705	9,646.4 (65.4)	–2.626 (0.022)	–3,673.4	–22,483.6	Absolutely dominated
(9) Hospital perspective 434 cases/year	69 (8)	2.19	0.963	28, 44, 17	631.4 (20.8)	0.111 (0.023)	+5,669.9	–87.0	Not cost-effective
(10) Daytime RATKA only, 312 cases/year	69 (8)	2.19	0.963	20, 31, 12	774.7 (20.3)	0.110 (0.024)	+7,048.4	–237.4	Not cost-effective

RATKA: robotic-assisted total knee arthroplasty, CC: capital cost, M: million, US$: United States Dollar, HR: hazard ratio for RATKA versus conventional total knee arthroplasty from the Weibull distribution, AL: aseptic loosening incidence during 4.5 years, aPJI: acute periprosthetic joint infection incidence during 4.5 years, cPJI: chronic periprosthetic joint infection incidence during 4.5 years, Incr. cost: grand average incremental cost, Incr. eff: grand average incremental effectiveness, QALYS: quality-adjusted life years, ICER: incremental cost-effectiveness ratio, INMB: incremental net monetary benefit

a capital costs overall and per individual represent those for the robot.

b quite close to being cost-effective.

c no direct non-medical costs.

### Setting and location

Patient-level data in this study was retrieved from Thailand, an upper-middle-income country with a mixed public–private healthcare system. Approximately 75% of the population utilizes public healthcare services, while 25% access private care. The Thai public healthcare system is built around the Universal Coverage Scheme (UCS), a government initiative that ensures access to essential health services for all citizens. The UCS is complemented by 2 other major public insurance schemes: the Civil Servant Medical Benefit Scheme (CSMBS) and the Social Security Scheme (SSS), which together form Thailand’s 3 main public healthcare programs.

In 2018, starting point of the data for this study, RATKA was restricted to a few high-volume tertiary academic hospitals owing to substantial capital and operating expenditures. This study was based at Siriraj Hospital, a leading public academic institution and the largest national referral tertiary care center affiliated with Mahidol University. Siriraj serves as a primary robotic training center in Thailand and represents a realistic implementation environment for RATKA within the public healthcare sector. This study examines explicitly the UCS system, which serves as the main healthcare coverage scheme for most Thai people, examining the cost-effectiveness of RATKA compared with COTKA in Thailand.

### Model assumptions

The model operated under several key assumptions. First, the maximum lifespan of patients was set to 110 years to ensure that the simulation did not yield biologically implausible ages. Second, we set a limit of 8 surgical interventions per patient for the treatment of TKA-related complications. Third, we focused solely on unilateral TKA and excluded all bilateral cases. Fourth, we employed adjusted capital costs in which the use of 1 robot was shared between knee and hip arthroplasty and between public and private patients. The capital costs for the base case analysis were adjusted to represent only knee arthroplasty performed in the public sector. Fifth, the model assumed the use of a robot similar to the “MAKO” system [23], which was assumed to be shared between hip and knee cases. The cost of this robot was adjusted by the ratio of knee usage for the model, derived from the hospital data. Other types of robots were not considered in this study. Sixth, all individuals in the model were assumed to follow the clinical care pathway of a tertiary hospital in Thailand. This pathway may differ from other settings and countries. For example, it did not include same-day surgery. Seventh, the model assumed that in the rare events such as those of salvage procedures (knee fusion, amputation, or resection arthroplasty), patients would either survive, die, or exit from the model without the possibility of repeated salvage procedures. Eighth, the costs of rare events were defined as fixed costs derived from the Thai standard cost list due to the lack of data on its distribution, range, and standard deviation in the dataset. Ninth, we focused only on individuals insured under the main health coverage scheme, “Universal Coverage” (UC) scheme, which guarantees all residents’ access to healthcare in Thailand [24,25].

### 2-dimensional simulation

The validated DES model was run as a 2-dimensional simulation consisting of sets of first-order iterations nested within each second-order iteration. A first-order iteration consisted of a single individual with characteristics sampled from individual-level distributions who traversed the RATKA strategy model structure and then restarted the simulation in the COTKA strategy. On each second-order iteration, samples were taken from population-level distributions. The sampled values were then used to simulate a set of first-order iterations (Supplementary Tables 2–4) [26-29]. First-order sample size was set at 5,425 to match the annual number of knee surgeries in the PM cohort (n = 434) multiplied by the 12.5 year estimated operational lifespan of a robot. Second-order sample size was determined empirically by increasing the number of iterations until average model outputs stabilized (n = 3,500) (Supplementary Figure 2). To simulate the Thai general population mortality, Weibull Time-to-Event (TTE) distribution parameters were estimated from the 2019 World Population Prospects mortality report by the United Nations [30] via a simulated annealing method [31]. The estimated gamma and lambda parameters were calculated separately for men and women at starting ages of 55, 60, 65, and 70 years to detail the expected mortality rates and derive life expectancy adjustments specific to each age range.

### Analysis plan in the simulation-model-based economic evaluation

#### Model outputs and cost-utility analysis

All aspects of model validation are defined in Supplementary Section 1. Clinical model outputs included overall survival and the numbers of primary complications such as the need for aseptic loosening, aPJI, cPJI, and the numbers of secondary surgeries or treatments to manage the 3 primary complications. Economic outcomes included the estimated difference in discounted, quality-adjusted lifespan (denominated in quality-adjust life years, QALYs) and the difference in estimated, discounted, total lifetime costs between the RATKA and COTKA strategies for each first-order iteration (individual). These differences were then averaged across the first-order iterations to yield the difference in estimated quality-adjusted life expectancy (QALE) and cost for a given second-order iteration. Grand averages over the second order iterations then used to estimate cost-utility measures: the incremental cost-effectiveness ratio (ICER = ΔCost/ΔQALE) and the incremental net monetary benefit (INMB = [ΔQALE * λ] – ΔCost) where λ is the Thai cost-effectiveness (CE) threshold of 160,000 Thai Baht or US$4,888/QALY gained [32]. RATKA would be estimated to be cost-effective relative to COTKA if the ICER is less than λ (which corresponds to a positive INMB). We generated incremental cost-effectiveness (ICE) plots depicting the ΔCost and ΔQALE for each second order iteration. On this plot, the λ threshold is represented as a dotted diagonal line. We also plotted cost-effectiveness acceptability curves (CEACs) depicting the proportion of second-order iterations that fell below the λ threshold for different values of λ.

#### Sensitivity analyses

2-dimensional simulation samples all model input parameters simultaneously and provides an indication of the degree to which ΔCost and ΔQALE values for the second-order iterations vary around the grand averages. We supplemented the latter with deterministic 1- and 2-way sensitivity analyses (DSAs) where 1 or 2 input parameters were varied, respectively, while all other model parameters were held fixed (Supplementary Table 5). All 1-way sensitivity analyses were displayed together as a Tornado diagram, which, from top to bottom, indicates the degree to which varying each input parameter affects the INMB.

#### Scenario analyses

For the base case analysis, we used the crude rate per patient-year of all complications combined for each primary TKA type from the PM cohort (Supplementary Tables 6–7). We also conducted 10 scenario analyses: 1—RATKA capital cost not adjusted by the proportion of public usage; 2—RATKA used at the maximum plausible cases/year (850) in public hospitals; 3—RATKA performed only in younger patients (55–65 years old); 4—RATKA performed only in older patients (65–70 years old); 5—Best plausible case for RATKA (capital cost US$611,000 and hazard ratio [HR] 0.6); 6—Worst plausible case for RATKA (capital cost US$6,110,000 and HR 0.975); 7—Extreme efficacy for RATKA (HR 0.024, estimated from the Weibull regression); 8—Extreme harm for RATKA (HR 41.71, estimated from the Weibull regression); 9—Hospital perspective; and 10—Daytime surgery only, limiting the number of TKA cases/year to 312 (Supplementary Table 8).

### Ethics, data sharing plan, funding, use of AI, and disclosures

The study was approved by the Institutional Review Board of Siriraj Hospital, Mahidol University, Thailand (COA: SI525/2022) to utilize patient-level data between January 2018 and June 2022. The Research Ethics Board of Sunnybrook Health Sciences Centre performed a delegated review and approved the study protocol (ID5641). The study was also approved by the Research Ethics Board Review of University of Toronto (RIS protocol number 43790). This study was not prospectively registered, as it is a model-based evaluation using retrospective data and does not involve an intervention. This study did not involve direct patient engagement in study design, outcome selection, or result interpretation

Individual-level data is kept at Siriraj Hospital database and requests for data should be directed to onlak.mak@mahidol.ac.th. Other inputs to the simulation model were derived from sources in the public domain, available in the open access repository, accessible at Github repository (github.com).

This study was unfunded. No external financial support was received.

Each author certifies that he has no commercial associations (e.g., consultancies, stock ownership, equity interest, patent/licensing arrangements, etc.) that might pose a conflict of interest in connection with the submitted article. Complete disclosure of interest forms according to ICMJE are available on the article page, doi: 10.2340/17453674.2025.44753

## Results

### Base-case analysis

**Figure 2:**
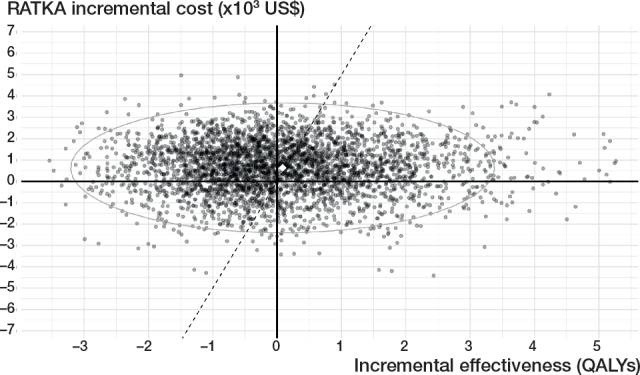
Incremental cost-effectiveness (ICE) scatter plot of the base-case analysis. US$: United States Dollar, QALYS: quality-adjusted life years.

**Figure 3:**
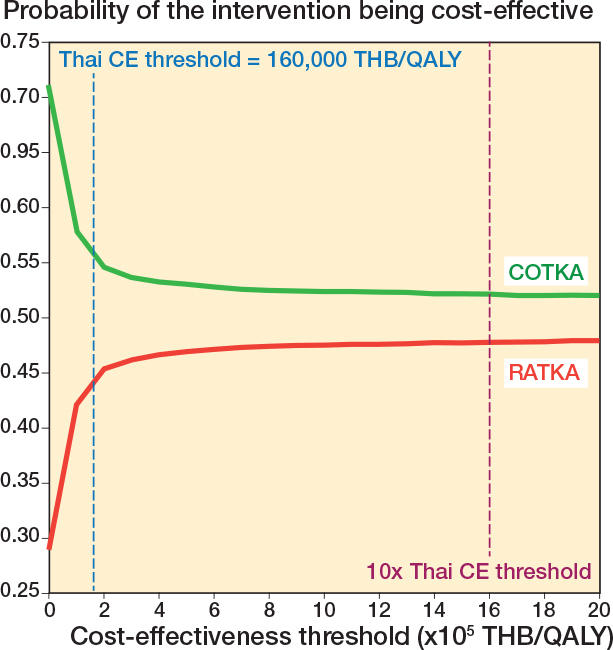
Cost-effectiveness acceptability curve (CEAC) of the base case analysis. CE: cost-effectiveness, COTKA conventional total knee arthroplasty QALY: quality-adjusted life year, RATKA: robotic-assisted total knee arthroplasty, THB: Thai Baht, US$: United States Dollar.

The base-case analysis estimated the grand average discounted lifetime cost per patient was US$5,031.90 for COTKA and US$5,666.86 for RATKA, with an overall ICER of US$7,435.62/QALY gained and an INMB of US$–217 with a grand average incremental cost of US$633.63 and a grand average incremental QALE of 0.085 QALY for RATKA relative to COTKA (Tables 1 and 2). The base-case results suggest RATKA is not expected to be cost-effective relative to COTKA at the Thai standard λ of US$4,888/QALY gained. However, the ICE plot showed a wide dispersion of second-order iterations around the grand averages (Figure 2). 36.3% of the iterations fell in the upper-left quadrant where RATKA was estimated to be more costly but generated lower QALE than COTKA. The CEAC demonstrated that the percentage of second-order iterations where RATKA was cost-effective at λ = 4,888 USD/QALY was 44.3% (Figure 3).

**Table 2:** Characteristics, estimated costs, effectiveness, and net monetary benefit results between 2 strategies. Values are count and mean (standard error)

Scenario type, RATKA annual cases	Sample sizes ^a^ 1st Oss	COTKA			RATKA		
		Costs ^b^ (US$)	Eff. (QALYs)	NMB (US$)	Costs ^b^ (US$)	Eff. (QALYs)	NMB (US$)
Base-case analysis							
Societal perspective, 434 cases/year	5,425	5,031.9 (17.7)	9.07 (0.02)	39,310.1 (99.4)	5,666.9 (11.6)	9.16 (0.01)	39,092.9 (64.4)
Scenario analyses							
(1) No adjustment for public/private usage, 434 cases/year	5,425	5,067.0 (17.7)	9.02 (0.02)	39,018.8 (101.1)	5,768.4 (11.6)	9.13 (0.01)	38,860.0 (63.4)
(2) Maximum robot usage, 850 cases/year	10,625	5,050.7 (17.6)	9.07 (0.02)	39,283.8 (98.0)	5,450.9 (11.5)	9.17 (0.01)	39,388.3 (62.03
(3) Younger patients, 434 cases/year	5,425	5,216.7 (18.2)	11.29 (0.02)	49,984.7 (119.0)	5,793.8 (11.2)	11.4 (0.02)	49,926.6 (78.9)
(4) Older patients, 434 cases/year	5,425	5,034.3 (18.0)	8.89 (0.02)	38,425.5 (96.9)	5,654.6 (11.8)	9.01 (0.01)	38,406.7 (62.7)
(5) Best plausible for RATKA, 434 cases/year	5,425	5,028.1 (17.4)	9.05 (0.02)	39,217.3 (98.3)	5,187.7 (11.3)	9.22 (0.01)	39,866.5 (65.1)
(6) Worst plausible for RATKA, 434 cases/year	5,425	5,040.0 (17.8)	9.02 (0.02)	39,029.8 (100.7)	6,406.4 (11.7)	9.13 (0.01)	38,247.5 (63.8)
(7) Extreme benefit for RATKA, 434 cases/year	5,425	5,026.9 (17.9)	9.00 (0.02)	38,989.7 (101.0)	5,150.9 (10.9)	9.27 (0.01)	40,178.2 (63.9)
(8) Extreme harm for RATKA, 434 cases/year	5,425	5,037.0 (17.8)	9.05 (0.02)	39,218.7 (97.3)	14,683.4 (67.3)	6.43 (0.01)	16,735.1 (92.6)
(9) Hospital perspective, **^c^** 434 cases/year	5,425	5,040.1 (18.0)	9.07 (0.02)	39,219.2 (97.9)	5,671.5 (11.5)	9.16 (0.01)	39,132.1 (63.7)
(10) Daytime RATKA only, 312 cases/year	3,900	5,030.4 (17.7)	9.06 (0.02)	39,142.4 (99.4)	5,805.1 (11.6)	9.17 (0.01)	38,905.0 (64.4)

RATKA: robotic-assisted total knee arthroplasty, US$: United States Dollar, 1st Oss: first-order iteration sample size, Cost: grand average cost, Eff: grand average effectiveness, QALYs: quality-adjusted life years, NMB: net monetary benefit.

a Second-order iteration sample size was 3,500 in all scenarios.

b All costs are discounted at 3% annually.

c no direct non-medical costs.

### Deterministic sensitivity analyses

**Figure 4:**
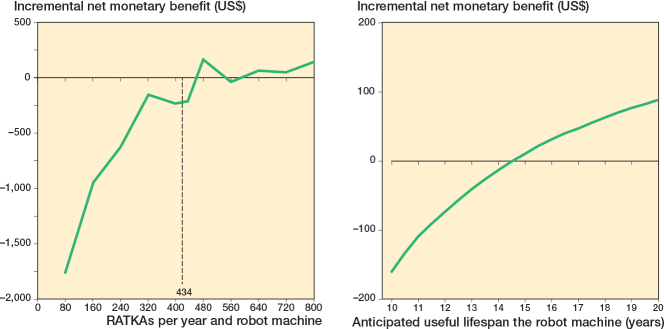
One-way sensitivity analysis (left panel) on surgical volume and (right panel) anticipated operational life span of a robot. RATKA: robotic-assisted total knee arthroplasty, US$: United States Dollar.

The Tornado diagram (Supplementary Figure 3) estimated that the 2 most influential input parameters were the HR for complications of RATKA vs COTKA and the capital cost of the robot. The undiscounted upfront capital cost threshold was US$2,653,374. This threshold reflects the maximum investment cost at which RATKA becomes cost-effective when all other variables are held at their mean values. The estimated INMB became positive above a surgical volume of 600 cases per year or if a robot’s operational lifespan was longer than 15 years (Figure 4).

**Figure 5:**
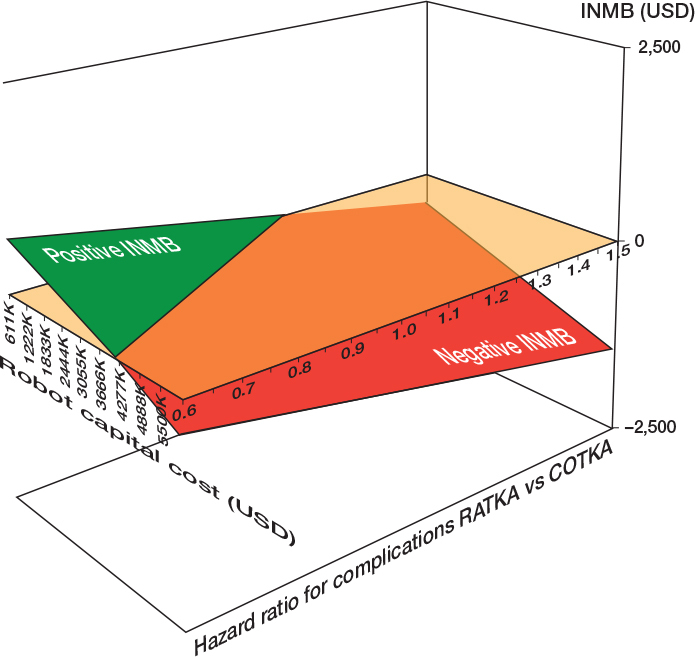
Two-way deterministic sensitivity analysis for combinations of robot capital cost and hazard ratio for the time to any complication after surgery for robotic-assisted total knee arthroplasty relative to conventional total knee arthroplasty. See Figure 3 for abbreviations and INMB: incremental net monetary benefit.

A 2-way DSA involving combinations of HR and capital cost illustrates combinations yielding a positive INMB (Figure 5 and Supplementary Table 8). In general, as either HRs or capital costs decreased, RATKA was estimated to be cost-effective relative to COTKA.

### Scenario analysis

From 10 different scenarios, RATKA was cost-effective in 3 specific cases. When the robot was utilized at its full capacity of 850 cases/year (scenario 2), it showed an ICER of US$3,876.0/QALY and a INMB of US$+104.6. Under the best plausible case for RATKA in terms of cost and efficacy (scenario 5), the ICER was US$965.0/QALY, and the INMB was US$+649.2. When RATKA yielded extreme efficacy in complication HR reduction (scenario 7), the ICER was US$461.7/QALY with an INMB of US$+1,188.4 (Tables 1 and 2).

## Discussion

We aimed to evaluate the cost-utility of RATKA vs COTKA from a societal perspective in Thailand, a MIC. Our base case analysis indicates that, under the MIC standard cost-effectiveness threshold, a broad adoption of RATKA within the public sector as a replacement for COTKA in treating patients with end-stage knee osteoarthritis is estimated to not be cost-effective overall. We note that the overall ICER was sensitive to the small incremental grand average QALE in the denominator for RATKA vs COTKA. In addition, there was substantial uncertainty among the second-order iterations surrounding the grand averages on the ICE plot. The latter was driven in part by the relatively few RATKA complications in our cohort and therefore substantial uncertainty regarding the reduction in the hazard of complications due to the use of a robot. As per the life-cycle approach to health technology assessment [33], this cost-effectiveness must therefore be considered preliminary in the early adoption phase of a new technology and potentially subject to revision once more data becomes available with respect to complication rates after RATKA.

In deterministic sensitivity analyses, we estimated that the cost-effectiveness of RATKA would be sensitive to 4 factors: the robot’s capital cost, its efficacy in terms of the HR for complications, its operational lifespan, and the annual surgical volume. Deterministic sensitivity analyses estimate that RATKA could achieve cost-effectiveness at high surgical volumes, either by increasing the lifespan of the robot or its operational capacity. In terms of the latter, cost effectiveness required at least 640 cases per year per 1 robot, which may result in implementation challenges. This volume requirement may be particularly hard to achieve in a setting where public healthcare is predominant.

Our study stands out as the first to evaluate the cost-effectiveness of RATKA using patient-level data in a MIC context. Previous model-based cost-utility analyses from high-income countries (HICs), such as the United States and Belgium, assessed RATKA using microsimulation models from a healthcare payer’s perspective, factoring in costs and quality-adjusted life expectancy (QALE) [10,11]. Both studies identified higher costs and QALE for RATKA but demonstrated cost-effectiveness primarily in higher-volume centers. The study from the United States suggested that RATKA was cost-effective in centers performing over 100 surgeries annually with ICERs under US$50,000/QALY. The Belgian study required an even higher threshold of 253 annual surgeries to achieve cost-effectiveness (Supplementary Table 9). Although our base-case estimates were qualitatively similar to the latter 2 studies, our conclusions differed primarily due to a much lower cost-effectiveness threshold in Thailand vs the United States or Belgium. More generally, our results support the notion that conclusions drawn from economic analyses in a HIC context may not directly apply to or provide realistic guidance for a MIC. Despite the absence of demonstrated clinical superiority, RATKA has been introduced in Thailand and is now increasingly gaining popularity, primarily through academic institutions and private sector investment. Our findings suggest that broader adoption in the public sector is not currently justified from a cost-effectiveness perspective. However, decisions around future implementation may also consider factors beyond economic efficiency—such as regional equity, long-term outcomes, and health system innovation.

From another viewpoint, our findings suggest that to achieve cost-effectiveness, robot manufacturers and health systems could focus on several key strategies: reducing the capital investment cost of the robot, improving its effectiveness especially in reducing postoperative complications, ensuring longer guaranteed operational lifespan at purchase, and enhancing the system’s software and equipment to improve surgical throughput. The latter, for instance, may involve innovations that shorten operative times and increase the number of daily procedures per robot. These insights may guide future procurement decisions and technology development priorities.

In a global perspective, the adoption of innovations in arthroplasty—including robotic-assisted surgery—has been influenced not only by economic factors but also by institutional readiness and surgeon familiarity with emerging techniques. As underlined in recent work [34], continuous exposure to these technological developments is critical for orthopedic improvement, especially in circumstances where the capacity to dramatically reduce short-term complications or cost-effectiveness is uncertain [6,7,10,11]. Our findings align with this view, highlighting the importance of early evaluation in MICs to support informed adoption pathways tailored to local health system priorities.

With regard to our findings, policy-makers have several options. The first would be to fund the procedure within the public sector regardless of the lack of cost-effectiveness because it might serve a particularly vulnerable group or somehow reduce inequities among the population with end-stage knee disease. Given that COTKA is a safe and effective procedure and that evidence of the clinical superiority of RATKA is weak, the latter argument is hard to make.

The second option would be for public administrators to take steps to increase the number of cases per operational year of the robot (e.g., by regionalizing orthopedic centers that provide RATKA), by extending the operational lifespan of each machine (perhaps even longer than recommended by the manufacturer), or by engaging in public–private partnerships to defray the expense incurred by the public sector. Regionalization may have equity issues: limiting the deployment of RATKA to high-volume centers may restrict access to this technology based on geographic location and disadvantage patients living further away from such centers [35]. Although referral systems may help mitigate access issues, this approach remains challenging given that the current clinical indications for RATKA are identical to those for COTKA, and explicit referral criteria have yet to be explored and defined in the future.

The third option would be to await further evidence regarding reduction in complication rates after RATKA from patients treated in the private sector and then repeat a cost-effectiveness analysis. A final option would be to conduct a randomized trial of RATKA vs COTKA in MIC. A preliminary step would be to conduct a value-of-information analysis to estimate the economic attractiveness of a trial to reduce uncertainty around the HR for complications of RATKA vs COTKA [36,37].

### Strengths

First, this study leveraged an advanced modelling technique combined with patient-level data, which offers many analytic advantages, including individual-level simulations that can track patients from their time of surgery to death or maximum age limit, allowing for realistic patient trajectories. This method avoids the discrete time biases commonly seen in other microsimulation models and enhances computational efficiency [38]. Second, the study utilized high-quality, real-world data from the largest RATKA training center in Thailand and a training hub in Southeast Asia. Third, the application of PM helped minimize the effect of selection biases in the data that informed the model inputs.

### Limitations

First, the patient-level input data was derived from a single high-volume public hospital and may not reflect other settings, such as private or lower-volume centers. Second, the assumption that outcomes of RATKA are comparable to existing literature from HICs may not hold due to economic and clinical practice differences. Third, the reliance on early-phase implementation data for RATKA, which only began in 2018, limits the long-term applicability of our findings. Given the nascent stage of robotic TKA in Thailand, our cost-effectiveness should be considered preliminary and may change as more data regarding complication rates after RATKA is accrued. Fourth, although the MAKO robot system used in this study has capabilities for partial knee replacement and potentially revision arthroplasty, our data included only its use for TKA and THA. Future work incorporating real-world utilization across broader indications may yield different cost-effectiveness thresholds and should be explored further.

### Conclusion

With the current best available evidence, a broad adoption of RATKA instead of COTKA into the public sector for knee osteoarthritis patients who have failed conservative treatment is not economically attractive given the cost-effectiveness threshold. This conclusion can be considered as preliminary given the current sparsity of data regarding the rate of complications post primary RATKA.

*In perspective,* our results raise questions regarding funding for technologies like RATKA in MIC settings, especially those under public health insurance schemes. This concern stems not from assumptions regarding clinical inferiority, but from the fundamental economic challenge faced by MICs context—limited healthcare budgets necessitate careful evaluation of whether expensive technologies such as RATKA deliver sufficient value for money in their specific context. Although previous evidence showed that robotic-assisted arthroplasty provided better radiographic accuracy, its effects on important clinical outcomes or ability to reduce revision surgery are still questionable [6,7] and its economic implications and feasibility within limited budgets necessitate a thorough consideration to ensure that health resources are distributed effectively and fairly. If longer-term evidence eventually demonstrates that RATKA substantially reduces revision rate, a re-evaluation of its cost-effectiveness would be warranted.

### Supplementary data

Supplementary Tables 1–9 and Supplementary Figures 1–3 are available as supplementary data on the article page, doi: 10.2340/17453674.2025.44753

## Supplementary Material



## Notes

PR initiated the research question, project administration, economic modelling, internal model validation, drafted, and finalized the manuscript and rebuttal. OR provided project administration on accessing institutional-level data, drafted, and finalized the manuscript and rebuttal. WI and MGZ performed face validation and external model validation and finalized the manuscript. DMJN conceptualized, face validated, undertook modelling, provided clinical-related advice, supervised the project, and finalized the manuscript.

The authors gratefully acknowledge Nichakorn Khomawut, for her cooperation; and Pattara Leelahavarong, Chaturong Pornrattanamaneewong, and Keerati Chareancholvanich for their unwavering support from Siriraj Hospital, Mahidol University, Thailand.

Handling co-editors: Li Felländer-Tsai and Robin Christensen

*Acta* thanks Birger Carl Forsberg for help with peer review of this manuscript.
