# Supplementary material for: Cost-effectiveness of robotic-assisted versus conventional total knee arthroplasty: an analysis from a middle income country
Source: Acta Orthop. 2025 Sep 30;96:716–25. doi: 10.2340/17453674.2025.44753 (PMC12489808; doi:10.2340/17453674.2025.44753)
Supplement: Supplementary file 1 [file ActaO-96-44753-s1.pdf]

Supplementary Table 1. CHEERS 2022 checklist

Topic	No.	Item	Location where item is reported
**Title**			
	1	Identify the study as an economic evaluation and specify the interventions being compared.	P. 1
**Abstract**			
	2	Provide a structured summary that highlights context, key methods, results, and alternative analyses.	Context P. 4<br>Method P.4<br>Results P.5<br>Alternative analyses P.5
**Introduction**			
**Background and objectives**	3	Give the context for the study, the study question, and its practical relevance for decision making in policy or practice.	P. 5-6
**Methods**			
**Health economic analysis plan**	4	Indicate whether a health economic analysis plan was developed and where available.	Indicate on P.7-10,<br>Model availability P.16,<br>Figure 1 – flow of study
**Study population**	5	Describe characteristics of the study population (such as age range, demographics, socioeconomic, or clinical characteristics).	P. 10
**Setting and location**	6	Provide relevant contextual information that may influence findings.	P. 11
**Comparators**	7	Describe the interventions or strategies being compared and why chosen.	Intervention P. 7,<br>All strategies P.15 (base case and scenario analyses)
**Perspective**	8	State the perspective(s) adopted by the study and why chosen.	P. 7
**Time horizon**	9	State the time horizon for the study and why appropriate.	P. 14,16 (lifetime)
**Discount rate**	10	Report the discount rate(s) and reason chosen.	P. 8

Topic	No.	Item	Location where item is reported
**Selection of outcomes**	11	Describe what outcomes were used as the measure(s) of benefit(s) and harm(s).	P. 14
**Measurement of outcomes**	12	Describe how outcomes used to capture benefit(s) and harm(s) were measured.	P. 14, 15
**Valuation of outcomes**	13	Describe the population and methods used to measure and value outcomes.	P. 14
**Measurement and valuation of resources and costs**	14	Describe how costs were valued.	P. 9-10, Supplementary Table 2
**Currency, price date, and conversion**	15	Report the dates of the estimated resource quantities and unit costs, plus the currency and year of conversion.	P. 10
**Rationale and description of model**	16	If modelling is used, describe in detail, and why used. Report if the model is publicly available and where it can be accessed.	Model details P. 7-8,<br>Model availability P.16<br>Supplementary Figure 1.1-1.11
**Analytics and assumptions**	17	Describe any methods for analysing or statistically transforming data, any extrapolation methods, and approaches for validating any model used.	Model assumption P. 12<br>Model Validation P. 13<br>Analysis details P.14
**Characterising heterogeneity**	18	Describe any methods used for estimating how the results of the study vary for subgroups.	P. 10, 14- 15
**Characterising distributional effects**	19	Describe how impacts are distributed across different individuals or adjustments made to reflect priority populations.	P. 10
**Characterising uncertainty**	20	Describe methods to characterise any sources of uncertainty in the analysis.	P. 10,<br>Supplementary Table 2,3,4,5
**Approach to engagement with patients and others affected by the study**	21	Describe any approaches to engage patients or service recipients, the general public, communities, or stakeholders (such as clinicians or payers) in the design of the study.	P. 16
**Results**			

Topic	No.	Item	Location where item is reported
**Study parameters**	22	Report all analytic inputs (such as values, ranges, references) including uncertainty or distributional assumptions.	P.8-10, Supplementary Table 2,3,4,5
**Summary of main results**	23	Report the mean values for the main categories of costs and outcomes of interest and summarise them in the most appropriate overall measure.	P. 17, Table 1, 2
**Effect of uncertainty**	24	Describe how uncertainty about analytic judgments, inputs, or projections affect findings. Report the effect of choice of discount rate and time horizon, if applicable.	P. 15, Figure 2,3,4,5 Supplementary Table 2,3,4,5 Supplementary Figure 3-effect of discount rate in Tornado diagram
**Effect of engagement with patients and others affected by the study**	25	Report on any difference patient/service recipient, general public, community, or stakeholder involvement made to the approach or findings of the study	P. 18, Table 1, Table 2 For all 10 scenario analyses
**Discussion**			
**Study findings, limitations, generalisability, and current knowledge**	26	Report key findings, limitations, ethical or equity considerations not captured, and how these could affect patients, policy, or practice.	P.18-22
**Other relevant information**			
**Source of funding**	27	Describe how the study was funded and any role of the funder in the identification, design, conduct, and reporting of the analysis	P.3 This is an unfunded study
**Conflicts of interest**	28	Report authors conflicts of interest according to journal or International Committee of Medical Journal Editors requirements.	P.3, All authors declare no conflicts of interest

Supplementary Table 2. Values and details of the cost inputs.

Item	Distribution type	Fixed value or distribution parameters	Data source
Total capital cost of robot	None	2.19 M USD (71.7 M THB)	Manufacturer
Up-front capital cost of robot (not discounted)	None	1.30 M USD (42.6 M THB)	Manufacturer
Annual maintenance costs of robot (discounted)	None	0.89 M USD (29.1 M THB)	Manufacturer
Aseptic revision knee arthroplasty after index surgery	Gamma	μ = 238<br>σ = 3,666.36 USD (120 K THB)	Patient-level data
Two-stage exchange for cPJI	Gamma	μ = 199<br>σ = 3,269.17 USD (107 K THB)	Patient-level data
Robotic-assisted total knee arthroplasty (RATKA)	Gamma	μ = 156<br>σ = 653.83 USD (21.4 K THB)	Patient-level data
Debridement, antibiotics, and implant retention (DAIR)	Gamma	μ = 148<br>σ = 1,750.69 USD (57.3 K THB)	Patient-level data
Conventional total knee arthroplasty (COTKA)	Gamma	μ = 148<br>σ = 1,032.69 USD (33.8 K THB)	Patient-level data
Intravenous antibiotics for superficial infection	Gamma	μ = 75.9<br>σ = 464.41 USD (15.2 K THB)	Patient-level data
Salvage procedure: knee arthrodesis	None	2147.88 USD (70.3 K THB)	Patient-level data
Salvage procedure: resection arthroplasty	None	1,420.71 USD (46.5 K THB)	Patient-level data
Salvage procedure: above knee amputation	None	840.21 USD (27.5 K THB)	Patient-level data
Hospitalization cost per day for aseptic revision	Gamma	μ = 10.2<br>σ = 239.23 USD (7.83 K THB)	Patient-level data
Hospitalization cost per day for COTKA	Gamma	μ = 6.00<br>σ = 54.69 USD (1.79 K THB)	Patient-level data
Hospitalization cost per day for RATKA	Gamma	μ = 5.64<br>σ = 51.33 USD (1.68 K THB)	Patient-level data
Hospitalization cost per day for acute infection post TKA	Gamma	μ = 5.06<br>σ = 80.35 USD (2.63 K THB)	Patient-level data
Hospitalization cost per day for cPJI	Gamma	μ = 3.22<br>σ = 73.33 USD (2.40 K THB)	Patient-level data
Hospitalization cost per day for intravenous antibiotics	Gamma	μ = 1.05<br>σ = 85.24 USD (2.79 K THB)	Patient-level data
Direct non-medical and indirect cost (per visit),	Gamma	μ = 0.558<br>σ = 4.46 USD (0.146 K THB)	Patient-level and HITAP

NOTE: μ – average value, σ – standard deviation, TKA – total knee arthroplasty, cPJI – chronic periprosthetic joint infection, USD – United States Dollar, M THB – millions Thai Baht, K THB – thousands of Thai Baht, HITAP – The Health Intervention and Technology Assessment Program.

Supplementary Table 3. Values and details of the utility inputs.

Health state	Distribution type	Fixed value or distribution parameters	Data source
Being well after RATKA	2-parameter beta distribution	m = 0.845, s = 0.070	Patient-level data
Being well after COTKA	2-parameter beta distribution	m = 0.835, s = 0.110	Patient-level data
Hospitalization after surgery	2-parameter beta distribution	m = 0.780, s = 0.180	Patient-level data
After aseptic revision	2-parameter beta distribution	m = 0.740, s = 0.150	Konopka JF, et al., 2018[a]
After septic revision	2-parameter beta distribution	m = 0.710, s = 0.220	Konopka JF, et al., 2018[a]
After knee disarticulation	None	0.6	Troendlin F, et al., 2020[b]
Before aseptic revision treatment	2-parameter beta distribution	m = 0.610, s = 0.200	Konopka JF, et al., 2018[a]
After above knee amputation	None	0.653	Hansson E, et al., 2018[c]
After knee arthrodesis	None	0.653	Hungerer S, et al., 2017[d]
Before septic TKA treatment	2-parameter beta distribution	m = 0.500, s = 0.300	Konopka JF, et al., 2018[a]

Notes: RATKA – robotic-assisted total knee arthroplasty, COTKA – conventional total knee arthroplasty, TKA – total knee arthroplasty, m – average value, s – standard deviation.

[a]Konopka JF, Lee Y yu, Su EP, McLawhorn AS. Quality-Adjusted Life Years After Hip and Knee Arthroplasty. *JB JS Open Access*. 2018;3(3):e0007. doi:10.2106/JBJS.OA.18.00007
bTroendlin F, Frieler S, Hanusrichter Y, Yilmaz E, Schildhauer TA, Baecker H. Persistent Fistula for

Treatment of a Failed Periprosthetic Joint Infection: Relic From the Past or a Viable Salvage Procedure? *J Arthroplasty*. 2020;35(2):544-549. doi:10.1016/j.arth.2019.09.012

[c]Hansson E, Hagberg K, Cawson M, Brodtkorb TH. Patients with unilateral transfemoral amputation treated with a percutaneous osseointegrated prosthesis: a cost-effectiveness analysis. *Bone Joint J*. 2018;100-B(4):527-534. doi:10.1302/0301-620X.100B4.BJJ-2017-0968.R1

[d]Hungerer S, Kiechle M, von Rüden C, Militz M, Beitzel K, Morgenstern M. Knee arthrodesis versus above-the-knee amputation after septic failure of revision total knee arthroplasty: comparison of functional outcome and complication rates. *BMC Musculoskelet Disord*. 2017;18(1):443. doi:10.1186/s12891-017-1806-8

Supplementary Table 4. Values and details of all miscellaneous variables (model input parameters and individual characteristics).

Item	Distribution type	Fixed value or distribution parameters	Data source
Sex (male = 1, female = 0)	Bernoulli	μ = 0.19	Patient-level data
Age at simulation start (base-case analysis, years)	Normal	μ = 69.1, σ = 7.87	Patient-level data
LOS RATKA (days)	4-parameter beta distribution	min =1, mode = 5, max = 13	Patient-level data
LOS COTKA (days)	4-parameter beta distribution	min =1, mode = 5, max = 17	Patient-level data
LOS DAIR (days)	4-parameter beta distribution	min =7, mode = 18, max = 36	Patient-level data
LOS IV antibiotics (days)	4-parameter beta distribution	min =14, mode = 18, max = 28	Patient-level data
LOS aseptic revision (days)	4-parameter beta distribution	min = 4, mode = 5, max = 7	Patient-level data
LOS two-stage revision (days)	4-parameter beta distribution	min = 7, mode = 11, max = 53	Patient-level data
Maximum number of revision surgeries (n)	Truncated Poisson	μ = 4, hard max = 8	Patient-level data
Gamma value for mortality starting age at 55 years	Weibull	Women = 1.93156287<br>Men = 1.700696258	WHO[a]
Gamma value for mortality starting age at 60 years	Weibull	Women = 1.710591294<br>Men = 1.495644499	WHO[a]
Gamma value for mortality starting age at 65 years	Weibull	Women = 1.510068218<br>Men = 1.360894816	WHO[a]
Gamma value for mortality starting age at 70 years	Weibull	Women = 1.37862775<br>Men = 1.225159496	WHO[a]
Lambda value for mortality starting age at 55 years	Weibull	Women = 0.001940079<br>Men = 0.005336776	WHO[a]
Lambda value for mortality starting age at 60 years	Weibull	Women = 0.005404188<br>Men = 0.014407257	WHO[a]
Lambda value for mortality starting age at 65 years	Weibull	Women = 0.013719537<br>Men = 0.027724655	WHO[a]
Lambda value for mortality starting age at 70 years	Weibull	Women = 0.026137639<br>Men = 0.057902752	WHO[a]

Note: μ – average value, σ – standard deviation, LOS – length of stay in hospital, RATKA – robotic-assisted total knee arthroplasty, COTKA – conventional total knee arthroplasty, DAIR – debridement, antibiotics and implant retention, min – minimum, mode – most likely value, max = maximum value

[a] United Nations, Department of Economic and Social Affairs, Population Division (2019). World Population Prospects 2019, Online Edition. Rev. 1.

Supplementary Table 5. Variables included in one-way deterministic sensitivity analyses (1-way DSAs).

Variable: sort by value	Base-case value	1-way DSA lowest value	1-way DSA highest value
1. Upfront capital cost (not discounted)	1,302,923.80	305,530.09	3,971,891.23
2. Annual capital cost (discounted)	888,453.53	305,530.09	2,138,710.66
3. Aseptic revision knee arthroplasty costs	7,275.26	3,600.76	10,949.75
4. Stage exchange for chronic PJI costs	6,078.31	2,818.33	9,338.30
5. Robotic-assisted total knee arthroplasty costs	4,752.42	4,099.04	4,514.88
6. Debridement, antibiotics, and implant retention costs	4,536.90	2,786.37	4,536.90
7. Conventional total knee arthroplasty costs	4,518.76	3,486.03	4,262.97
8. Intravenous antibiotic for superficial infection costs	2,320.79	1,856.64	2,320.79
9. Hospitalization cost per day for aseptic revision	312.16	73.04	551.29
10. Hospitalization cost per day for COTKA	183.17	128.38	237.96
11. Hospitalization cost per day for RATKA	172.23	120.93	223.54
12. Hospitalization cost per day for acute infection post-TKA	154.51	74.04	234.97
13. Hospitalization cost per day for chronic PJI	98.25	25.04	171.45
14. Hospitalization cost per day for intravenous antibiotics	32.11	21.39	117.37
15. Direct non-medical cost	17.04	12.57	61.11
16. Starting age	69.06	55.00	70.00
17. Years of anticipated use of RATKA	12.5	10	20
18. Hazard ratio for complications of RATKA vs COTKA	0.963	0.6	0.975
19. Utility of being well after RATKA	0.845	0.775	0.915
20. Utility of being well after COTKA	0.835	0.730	0.945
21. Utility of hospitalization after COTKA	0.780	0.630	0.930
22. Utility after aseptic revision	0.740	0.560	0.740
23. Utility after septic revision	0.710	0.490	0.710
24. Utility before aseptic revision treatment	0.610	0.410	0.810
25. Utility before septic TKA treatment	0.500	0.200	0.800

NOTE: Columns give the value used in the base case analysis and the lower and upper bounds, respectively, for the range of values employed in the DSAs. Cost unit were USD.

RATKA – robotic-assisted total knee arthroplasty, COTKA – conventional total knee arthroplasty, TKA – total knee arthroplasty

Supplementary Table 6. Summary of baseline demographics among the pre and post propensity-matched population.

Variables	Pre-matching (R=157, C=2253)				Post-matching (R=157, C=1570)			
	RATKA mean (SD)	COTKA mean (SD)	Standardized difference	t-test p-value	RATKA Mean (SD)	COTKA mean (SD)	Standardized difference	t-test p-value
Age	69.7(8.17)	69.1(7.95)	0.02	0.33	69.72(8.17)	69.02(7.88)	0.09	0.31

Variables	Pre-matching (R=157, C=2253)						Post-matching (R=157, C=1570)					
	RATKA		COTKA		Standardized difference	χ^2 p-value	RATKA		COTKA		Standardized difference	χ^2 p-value
	n	%	n	%			n	%	n	%		
Male sex	34	21.6	333	14.78	0.18	0.03	34	21.66	298	18.98	0.07	0.48
Metastatic cancer	0	6	1	0.04	0.07	1.00	0	0	0	0	NA	NA
Depression	1	0	24	1.07	0.05	0.92	1	0.64	14	0.89	0.03	1.00
Alzheimer's disease	1	0.64	8	0.36	0.04	1.00	1	0.64	2	0.13	0.08	0.65
Parkinson's disease	2	0.64	26	1.15	0.01	1.00	2	1.27	16	1.02	0.02	1.00
Diabetes mellitus	15	1.27	470	20.86	0.31	0	15	9.55	150	9.55	0	1.00
Chronic kidney disease	4	9.55	44	1.95	0.04	0.83	4	2.55	27	1.72	0.06	0.67
Obesity	0	2.55	16	0.71	0.12	0.58	0	0	12	0.76	0.12	0.55
Pulmonary disease	1	0	5	0.22	0.06	0.86	1	0.64	4	0.25	0.06	0.94
Cardiovascular disease	1	0.64	2	0.09	0.09	0.48	1	0.64	2	0.13	0.08	0.65

Note: Propensity matching did provide covariate balance but reduced the number of complications available for subsequent analyses.

R or RATKA – robotic-assisted total knee arthroplasty, C or COTKA – conventional total knee arthroplasty, SD – standard deviation.

Supplementary Table 7. Summary of complications among the pre and post propensity-matched population.

Complications	Pre-matching (R=157, C=2253)			Post-matching (R=157, C=1570)		
	AL	aPJI	cPJI	AL	aPJI	cPJI
Event in C (n)	17	33	8	0	1	0
Event in R (n)	7	13	4	0	1	0

Note: Propensity matching did provide covariate balance but reduced the number of complications available for subsequent analyses.

R– robotic-assisted total knee arthroplasty, C– conventional total knee arthroplasty, AL – aseptic loosening, aPJI – acute periprothetic joint infection, cPJI – chronic periprosthetic joint infection

Supplementary Table 8. Incremental net monetary benefit (INMB) from each combination of hazard ratio and capital cost.

HR	20M THB 611K USD	30M THB 917K USD	40M THB 1222K USD	50M THB 1528K USD	60M THB 1833K USD	70M THB 2138K USD	80M THB 2444K USD	90M THB 2749K USD	100M THB 3055K USD	110M THB 3361K USD	120M THB 3666K USD	130M THB 3972K USD	140M THB 4277K USD	150M THB 4538K USD	160M THB 4888K USD	170M THB 5194K USD	180M THB 5500K USD	190M THB 5805K USD	200M THB 6111K USD
1.500	-408.35	-464.67	-520.98	-577.30	-633.62	-689.94	-746.26	-802.58	-858.90	-915.22	-971.54	-1027.85	-1084.17	-1140.49	-1196.81	-1253.13	-1309.45	-1365.77	-1422.09
1.475	-379.59	-435.91	-492.23	-548.54	-604.86	-661.18	-717.50	-773.82	-830.14	-886.46	-942.78	-999.10	-1055.41	-1111.73	-1168.05	-1224.37	-1280.69	-1337.01	-1393.33
1.450	-347.06	-403.38	-459.70	-516.02	-572.34	-628.65	-684.97	-741.29	-797.61	-853.93	-910.25	-966.57	-1022.89	-1079.21	-1135.52	-1191.84	-1248.16	-1304.48	-1360.80
1.425	-315.85	-372.17	-428.49	-484.81	-541.13	-597.45	-653.77	-710.09	-766.40	-822.72	-879.04	-935.36	-991.68	-1048.00	-1104.32	-1160.64	-1216.96	-1273.28	-1329.59
1.400	-288.18	-344.50	-400.82	-457.14	-513.46	-569.78	-626.10	-682.41	-738.73	-795.05	-851.37	-907.69	-964.01	-1020.33	-1076.65	-1132.97	-1189.28	-1245.60	-1301.92
1.375	-258.25	-314.57	-370.89	-427.21	-483.53	-539.85	-596.17	-652.49	-708.81	-765.12	-821.44	-877.76	-934.08	-990.40	-1046.72	-1103.04	-1159.36	-1215.68	-1271.99
1.350	-231.31	-287.63	-343.95	-400.27	-456.59	-512.91	-569.22	-625.54	-681.86	-738.18	-794.50	-850.82	-907.14	-963.46	-1019.78	-1076.10	-1132.41	-1188.73	-1245.05
1.325	-201.03	-257.35	-313.67	-369.99	-426.31	-482.63	-538.95	-595.26	-651.58	-707.90	-764.22	-820.54	-876.86	-933.18	-989.50	-1045.82	-1102.13	-1158.45	-1214.77
1.300	-172.39	-228.71	-285.03	-341.35	-397.67	-453.99	-510.31	-566.63	-622.95	-679.27	-735.58	-791.90	-848.22	-904.54	-960.86	-1017.18	-1073.50	-1129.82	-1186.14
1.275	-144.67	-200.99	-257.31	-313.62	-369.94	-426.26	-482.58	-538.90	-595.22	-651.54	-707.86	-764.18	-820.49	-876.81	-933.13	-989.45	-1045.77	-1102.09	-1158.41
1.250	-117.04	-173.36	-229.67	-285.99	-342.31	-398.63	-454.95	-511.27	-567.59	-623.91	-680.23	-736.54	-792.86	-849.18	-905.50	-961.82	-1018.14	-1074.46	-1130.78
1.225	-90.91	-147.23	-203.55	-259.86	-316.18	-372.50	-428.82	-485.14	-541.46	-597.78	-654.10	-710.42	-766.74	-823.05	-879.37	-935.69	-992.01	-1048.33	-1104.65
1.200	-64.93	-121.25	-177.57	-233.89	-290.21	-346.52	-402.84	-459.16	-515.48	-571.80	-628.12	-684.44	-740.76	-797.08	-853.39	-909.71	-966.03	-1022.35	-1078.67
1.175	-35.73	-92.05	-148.37	-204.69	-261.01	-317.33	-373.65	-429.97	-486.29	-542.60	-598.92	-655.24	-711.56	-767.88	-824.20	-880.52	-936.84	-993.16	-1049.48
1.150	-0.46	-56.78	-113.10	-169.42	-225.73	-282.05	-338.37	-394.69	-451.01	-507.33	-563.65	-619.97	-676.29	-732.60	-788.92	-845.24	-901.56	-957.88	-1014.20
1.125	29.04	-27.28	-83.60	-139.92	-196.24	-252.56	-308.88	-365.19	-421.51	-477.83	-534.15	-590.47	-646.79	-703.11	-759.43	-815.75	-872.06	-928.38	-984.70
1.100	63.05	6.73	-49.59	-105.91	-162.23	-218.55	-274.86	-331.18	-387.50	-443.82	-500.14	-556.46	-612.78	-669.10	-725.42	-781.74	-838.05	-894.37	-950.69
1.075	91.02	34.70	-21.61	-77.93	-134.25	-190.57	-246.89	-303.21	-359.53	-415.85	-472.17	-528.48	-584.80	-641.12	-697.44	-753.76	-810.08	-866.40	-922.72
1.050	123.48	67.16	10.84	-45.48	-101.79	-158.11	-214.43	-270.75	-327.07	-383.39	-439.71	-496.03	-552.35	-608.67	-664.98	-721.30	-777.62	-833.94	-890.26
1.025	151.38	95.06	38.74	-17.58	-73.90	-130.21	-186.53	-242.85	-299.17	-355.49	-411.81	-468.13	-524.45	-580.77	-637.08	-693.40	-749.72	-806.04	-862.36
1.000	179.20	122.88	66.56	10.24	-46.08	-102.40	-158.72	-215.04	-271.35	-327.67	-383.99	-440.31	-496.63	-552.95	-609.27	-665.59	-721.91	-778.22	-834.54
0.975	203.18	146.86	90.54	34.23	-22.09	-78.41	-134.73	-191.05	-247.37	-303.69	-360.01	-416.33	-472.64	-528.96	-585.28	-641.60	-697.92	-754.24	-810.56
0.950	232.32	176.00	119.68	63.36	7.04	-49.28	-105.59	-161.91	-218.23	-274.55	-330.87	-387.19	-443.51	-499.83	-556.15	-612.46	-668.78	-725.10	-781.42
0.925	259.48	203.16	146.84	90.52	34.21	-22.11	-78.43	-134.75	-191.07	-247.39	-303.71	-360.03	-416.35	-472.66	-528.98	-585.30	-641.62	-697.94	-754.26
0.900	286.37	230.05	173.73	117.41	61.09	4.77	-51.55	-107.87	-164.18	-220.50	-276.82	-333.14	-389.46	-445.78	-502.10	-558.42	-614.74	-671.05	-727.37
0.875	318.78	262.46	206.15	149.83	93.51	37.19	-19.13	-75.45	-131.77	-188.09	-244.41	-300.72	-357.04	-413.36	-469.68	-526.00	-582.32	-638.64	-694.96
0.850	343.23	286.91	230.59	174.27	117.95	61.64	5.32	-51.00	-107.32	-163.64	-219.96	-276.28	-332.60	-388.92	-445.24	-501.55	-557.87	-614.19	-670.51
0.825	371.47	315.15	258.83	202.51	146.20	89.88	33.56	-22.76	-79.08	-135.40	-191.72	-248.04	-304.36	-360.67	-416.99	-473.31	-529.63	-585.95	-642.27
0.800	402.85	346.53	290.21	233.89	177.57	121.25	64.93	8.62	-47.70	-104.02	-160.34	-216.66	-272.98	-329.30	-385.62	-441.94	-498.25	-554.57	-610.89
0.775	434.13	377.82	321.50	265.18	208.86	152.54	96.22	39.90	-16.42	-72.74	-129.05	-185.37	-241.69	-298.01	-354.33	-410.65	-466.97	-523.29	-579.61
0.750	469.88	413.56	357.24	300.92	244.60	188.28	131.96	75.64	19.33	-36.99	-93.31	-149.63	-205.95	-262.27	-318.59	-374.91	-431.23	-487.54	-543.86
0.725	500.42	444.10	387.78	331.46	275.14	218.82	162.50	106.19	49.87	-6.45	-62.77	-119.09	-175.41	-231.73	-288.05	-344.37	-400.68	-457.00	-513.32
0.700	526.05	469.73	413.41	357.09	300.78	244.46	188.14	131.82	75.50	19.18	-37.14	-93.46	-149.78	-206.09	-262.41	-318.73	-375.05	-431.37	-487.69
0.675	557.79	501.47	445.15	388.84	332.52	276.20	219.88	163.56	107.24	50.92	-5.40	-61.72	-118.03	-174.35	-230.67	-286.99	-343.31	-399.63	-455.95
0.650	593.34	537.02	480.70	424.38	368.06	311.74	255.42	199.10	142.78	86.47	30.15	-26.17	-82.49	-138.81	-195.13	-251.45	-307.77	-364.09	-420.40
0.625	623.99	567.67	511.35	455.03	398.71	342.39	286.07	229.75	173.44	117.12	60.80	4.48	-51.84	-108.16	-164.48	-220.80	-277.12	-333.43	-389.75
0.600	652.07	595.75	539.43	483.11	426.80	370.48	314.16	257.84	201.52	145.20	88.88	32.56	-23.76	-80.08	-136.39	-192.71	-249.03	-305.35	-361.67

Note: Color code yellow = base case analysis range, light red = negative incremental net monetary benefit, light green = positive incremental net monetary benefit, M THB – million Thai Baht, K USD – Thousand United States Dollar

Supplementary Table 9. Characteristics and outputs of the previous economic evaluations compared to ours.

Details	Vermue 2021[a]	Rajan 2022[b]	Current study
**Study Setting, Conflict of Interest**	Belgium (High income country), no conflict of interest	USA (High income country), conflict of interest with industry for some authors	Thailand (Low to middle income country), no conflict of interest
**Population**	Patients 67 years old with primary OA knee undergoing TKA	Patients 60 years old with advanced degenerative knee undergoing TKA	Base case: mean 69 years old with primary OA knee undergoing unilateral TKA, with 10 scenarios analyses.
**Robot System**	MAKO (Stryker, USA)	Not reported	MAKO (Stryker, USA)
**Cost Perspective**	Cost-utility,<br>healthcare payer	Cost-utility,<br>healthcare payer	Cost-utility, both societal and hospital perspectives
**Cost year, currency**	Not reported, US dollar	2020, US dollar	2018-2022, USD converted from THB (32.73THB=1USD)
**Cost (converted to THB)**	RATKA vs. COTKA<br>40,427 USD vs.<br>29,035 USD	COTKA: 25,113 USD<br>RATKA: Low volume -92,823 USD<br>Mid volume - 29,261 USD<br>High volume – 25,730 USD	COTKA: 5,032.12 USD (164,701.34 THB)<br>RATKA: 5,665.77 USD (185,440.52 THB)
**Model structure**	Microsimulation model with patient-level health state transitions	Microsimulation model with patient-level health state transitions	Discrete event simulation with 2D simulation
**Model time**	Discrete,<br>cycle length = 1 year	Discrete,<br>cycle length = 1 year	Continuous, time units = years
**Discrete time bias correction**	Not stated	Not stated	Unnecessary given use of continuous time
**Treatment effect and outcome measures**	Quality well-being index score, QALYs	COTKA: summarized estimates from many QOL values, QALYs<br>RATKA: SF-36, QALYs	EQ5D5L converted to utility, QALYs
**Sources of Data**	Clinical: revision rate based on evidence of improved alignment with RATKA.<br>Cost: Ferket et al.'s study and data from Stryker. Utility: a study by Slover et al. (1356 patients)	Clinical: 11 studies published between 2007 and 2020.<br>Cost: Medicare reimbursement data.<br>Utility: similar cost-effectiveness studies	Clinical: patient-level data from 2018 to 2022 (N = 3,149, post-propensity match N=1,752),<br>WHO report 2019, literature<br>Cost: Patient-level data, HITAP standard cost list for Thailand<br>Utility: Patient-level data, literature
**Main Assumptions and Remarks**	Knee malalignment leads to an equal rate of increased revision in both groups, one revision cycle	Fixed cost of robotic surgery, one revision cycle, quality of life of all revisions the same, societal costs and quality of life of suboptimal TKA the same for both interventions	Focus on tertiary public utilization from societal perspective, 12.5 years of anticipated robot use in which 5,425 patients could benefit, no simulated patients live longer than 110 years, multiple revision cycles allowed with mean of 4 and maximum of 8, one robot's shared use between knee and hip arthroplasty and public per private sector accounted for in the adjusted capital costs.

**Time horizon, discounting**	20 years, discount rate 3%	Lifetime, discount rate 3%	Lifetime, discount rate 3 %
**Effectiveness**	RATKA vs. COTKA QALYs: 9.22 vs. 9.16	RATKA vs. COTKA QALYs: 13.55 vs. 13.29	RATKA vs COTKA QALYs: 9.15595 vs 9.07074
**CUA summary statistics**	ICERS ($/QALY) Mid volume (70/y): $376,145 High volume (253/y): <$50,000	ICERS ($/QALY) Low volume (13/y): $92,823 Mid volume (100/y): $29,261 High volume (200/y): $25,730	INMB (USD): -217.11 (-7,106.04 THB),<br>ICER (USD/QALY): 7435.62 (+243,397.20 THB/QALY) (434/y)<br>One robot machine must handle more than 640 cases per year to become cost-effective
**Sensitivity analyses**	- utilities and primary surgery success (~revision rate) had the biggest impact on ICERs - probabilistic: at $50,000 CE threshold, RATKA was cost-effective 2.18% of the time	-If the annualized revision rate for COTKA is 0%, RATKA would still be preferred at $100,000/QALY. RATKA revision rate would have to increase from 0.6% to 1.6% or 2.2% for COTKA to be preferred at $50,000 and $100,000/QALY, respectively – probabilistic: at $50,000 and $100,000/QALY, RATKA was cost-effective 67.5% and 68.5% of the time	- Deterministic<br>one-way and two-way– hazard ratio and capital cost have the highest influence on the model output.<br><br>-Probabilistic: at Thai λ, RATKA is cost-effective 44.34% of the time.

Note:

[a] Vermue H, Tack P, Gryson T, Victor J. Can robot-assisted total knee arthroplasty be a cost-effective procedure? A Markov decision analysis. Knee. 2021;29:345-352. doi:10.1016/j.knee.2021.02.004

[b] Rajan PV, Khlopas A, Klika A, Molloy R, Krebs V, Piuzzi NS. The Cost-Effectiveness of Robotic-Assisted Versus Manual Total Knee Arthroplasty: A Markov Model–Based Evaluation. JAAOS - Journal of the American Academy of Orthopaedic Surgeons. 2022;30(4):168-176. doi:10.5435/JAAOS-D-21-00309
